# Psychological Outcomes After Hypospadias Repair: Does Age at Surgery Matter? A Systematic Review

**DOI:** 10.7759/cureus.109964

**Published:** 2026-05-31

**Authors:** Randy Fauzan, Irfan Wahyudi, Gerhard Reinaldi Situmorang, Arry Rodjani, Putu Angga Risky Raharja

**Affiliations:** 1 Urology, Rumah Sakit Umum Pusat Nasional Dr. Cipto Mangunkusumo, Jakarta, IDN

**Keywords:** hypospadias repair, patient age, psychological outcomes, psychosexual development, systematic review

## Abstract

Hypospadias represents one of the most frequent congenital anomalies in male neonates, and corrective surgery is generally advised within the first 6 to 18 months of life. Nevertheless, the most appropriate time to perform the repair remains debated, especially concerning its impact on patients’ long-term psychological well-being. The present systematic review evaluated how the age at which surgery is performed affects subsequent psychological and psychosexual outcomes. A structured search of PubMed, ScienceDirect, Embase, and Cochrane Library was undertaken in May 2025, following the Preferred Reporting Items for Systematic Reviews and Meta-Analyses (PRISMA) 2020 reporting framework. Eligible studies were comparative investigations and cohorts that reported psychiatric, psychological, or psychosexual outcomes after primary hypospadias correction in pediatric populations. Two independent reviewers appraised risk of bias with the Newcastle-Ottawa Scale, and the Grading of Recommendations, Assessment, Development and Evaluations (GRADE) framework was applied to assess the certainty of evidence. As substantial heterogeneity precluded statistical pooling, a narrative synthesis was conducted. A total of 15 studies (more than 10,000 patients), published between 1997 and 2025 and originating from Europe, Asia, the Middle East, Australia, and North America, were ultimately included. The majority of patients who underwent hypospadias repair reached adequate psychosocial functioning in the long term, although a recurring subgroup demonstrated symptoms of anxiety, depression, obsessive-compulsive features, or dissatisfaction with body image. Large registry-based cohorts indicated heightened susceptibility to neurodevelopmental and psychiatric conditions, whereas smaller institutional series tended to highlight concerns related to genital self-perception. Whether surgical timing meaningfully alters these outcomes was not consistently supported across studies. Overall certainty of the evidence ranged from low to moderate. Taken together, psychological outcomes following hypospadias correction tend to be favorable, but the role of operative age in shaping long-term psychosocial well-being remains unclear. Well-designed prospective investigations with extended follow-up are warranted to address this question.

## Introduction and background

Hypospadias is a developmental disorder of the male external genitalia in which the urethral folds fail to fuse completely during fetal life, leaving the urethral opening positioned on the ventral aspect of the penis. The malformation is frequently accompanied by a dorsally hooded prepuce, downward penile curvature, and, when severe, a split scrotum or genitalia with an ambiguous appearance. Reported birth prevalence is roughly one affected boy in every three hundred live male births, ranking it among the most prevalent congenital genitourinary anomalies [1,2]. Operative reconstruction seeks to restore a functioning urethra, achieve a normally positioned meatus, and produce a straight, aesthetically acceptable penis. Multiple factors are weighed when selecting the timing of operation, encompassing developmental milestones, psychological readiness, anesthetic safety, and technical considerations [1,3].

Performing surgery at younger ages may increase exposure to anesthetic hazards, including respiratory or cardiovascular events. Conversely, postponing the procedure raises concerns about the patient’s psychological development, prompting some commentators to suggest that operations on the genitalia should be delayed until the child can give informed assent [4]. Current professional bodies, including the American Academy of Pediatrics and major European societies, advise that hypospadias correction be carried out between six and eighteen months of age. These recommendations, however, rest primarily on level 4 expert consensus and well-conducted level 3 observational research rather than randomized trial evidence [5].

Even with these consensus guidelines, real-world practice shows wide international variability driven by healthcare access, cultural views, and parental decision-making [6]. The controversy endures because operating very early entails anesthetic risk, whereas late repair has been associated with greater surgical complication rates and unfavorable psychosocial sequelae [7,8]. Available psychosexual data indicate that, although most operated individuals reach satisfactory adjustment, a persistent subset reports anxiety, depression, or unhappiness with genital appearance [9-11]. Because the relationship between operative age and these specific psychological domains remains insufficiently characterized, a systematic appraisal is justified. The aim of this review is, therefore, to consolidate available evidence and clarify how surgical timing relates to long-term psychological outcomes after hypospadias correction.

## Review

Methods

*Eligibility Criteria* 

Studies were judged eligible for inclusion if they satisfied the following requirements: (1) randomized controlled trials (RCTs) or comparative non-randomized studies (NRSs) comparing patient age groups in relation to psychological outcomes after hypospadias repair; (2) full-text articles published in English (English-language restriction was applied; this constitutes a recognized potential source of language bias); (3) patients undergoing primary repair of hypospadias; (4) all hypospadias surgical types and techniques accepted; and (5) at least one psychological or psychiatric outcome reported, including psychosexual development, behavioral changes, anxiety, depression, or neurodevelopmental disorders. Excluded from the review were single case reports, case series, preclinical animal investigations, conference abstracts without full text, narrative review articles, and previously published meta-analyses.

*Information Sources and Search Strategy* 

Reporting of this work follows the recommendations set out in the updated 2020 Preferred Reporting Items for Systematic Reviews and Meta-Analyses (PRISMA) statement and the methodology described in the Cochrane Handbook [12,13]. Four databases, PubMed, ScienceDirect, Embase, and the Cochrane Library, were queried in May 2025, limited to English-language publications without date restriction. Prior to commencement, the protocol was lodged in PROSPERO (CRD420261391631). Search syntax was tailored per database using the following concept clusters: (patient age OR timing of hypospadias repair) AND (psychological aspects OR cognitive development OR genital awareness OR emotional development OR psychosexual development) AND (hypospadias repair OR hypospadias surgery OR urethroplasty). No restriction was placed on the year of publication. The complete, database-specific search strings as executed across all four databases, including all Boolean operators, MeSH terms, and field tags, are provided in full in Supplementary Appendix A to ensure reproducibility.

Selection Process and Data Extraction** **

Eligible studies were identified within the conventional Population, Intervention, Comparison, Outcomes, and Study design (PICOS) framework. Screening of titles, abstracts, and full-text articles against the prespecified eligibility criteria was performed independently by two reviewers, and any disagreements were settled by consensus through discussion. Inter-reviewer agreement was not formally quantified using kappa statistics; however, all discrepancies at both the screening and data extraction stages were identified and resolved through structured discussion between the two independent reviewers until consensus was reached, consistent with Cochrane Handbook recommendations. In situations where the same study population had been reported in more than one publication, only the report containing the most comprehensive data was kept. Data extraction was carried out in duplicate by two reviewers working independently, capturing the following items: study design, publication year, country, total sample size, age categories at the time of operation, psychological outcomes reported, and the measurement instruments employed.

*Risk of Bias and Certainty of Evidence* 

Each included study was independently appraised by both reviewers using the Newcastle-Ottawa Scale (NOS) [14], which scores observational studies across three quality dimensions: cohort selection, comparability of groups, and ascertainment of the outcome. To convey the strength of the overall evidence for each outcome, the Grading of Recommendations, Assessment, Development and Evaluations (GRADE) framework was subsequently applied [15].

Synthesis and Reporting Bias

Owing to marked variability across studies in design, populations, and instruments used, statistical pooling was not pursued. A narrative synthesis was therefore undertaken and organized according to study design, age at operation, and category of outcome. Where available, standardized psychometric measures or registry-derived diagnoses were summarized. Because fewer than 10 studies addressed any single outcome, formal evaluation of reporting bias via funnel plots or related statistical methods was not feasible; the potential influence of unreported negative or null results was instead considered qualitatively.

Results of the review

Study Selection

**Figure 1:**
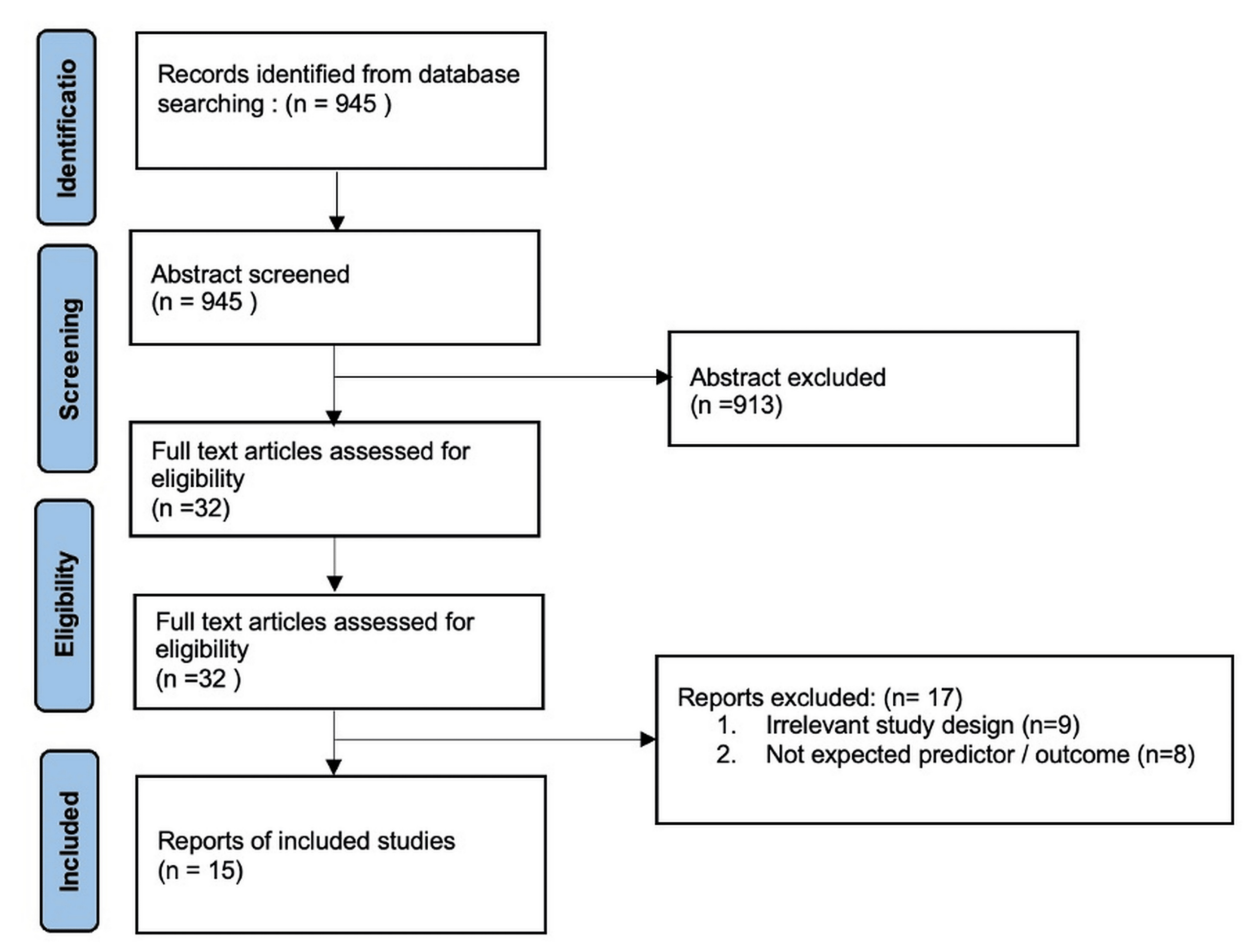
PRISMA 2020 flow diagram detailing literature identification, screening, eligibility assessment, and final study inclusion.

The database queries returned 945 records overall. After removal of duplicates and screening of titles and abstracts, 32 publications proceeded to full-text evaluation. Of these, 17 were rejected: nine for an unsuitable study design and eight for the absence of the predictor or outcome of interest. Fifteen studies satisfied all inclusion criteria and were entered into the qualitative synthesis (Figure 1).

*Study Characteristics* 

Publications meeting our inclusion criteria appeared between 1997 and 2025, and their designs spanned the spectrum of observational research: retrospective and prospective cohort investigations, case-control work, cross-sectional surveys, and large analyses based on national registries. Source countries spanned European, Asian, Middle Eastern, Australasian, and North American settings, and enrolled populations ranged broadly, from under 70 patients in some single-institution series to well over 9000 in the largest registry analyses. The reported age at corrective surgery varied substantially across studies; several explicitly contrasted operations performed early in life (younger than 18 months or younger than 2 years) with those carried out later, whereas other reports emphasized long-term follow-up extending into adolescence or adult life. The endpoints captured across the included studies encompassed psychosexual maturation, behavioral disturbances, anxious and depressive symptomatology, obsessive-compulsive disorder (OCD), attention-deficit/hyperactivity disorder (ADHD), autism spectrum disorder (ASD), and broader psychiatric morbidity. Outcome measurement strategies varied widely, encompassing structured clinical interviews, registry-derived diagnostic codes, and validated self- and parent-report questionnaires, such as the child behavior checklist (CBCL) and youth self-report (YSR) [16], pediatric quality of life inventory (PedsQL) [17], strengths and difficulties questionnaire (SDQ) [18], children's depression inventory (CDI) [19], revised child anxiety and depression scale (RCADS) [20], Zung self-rating depression scale (Zung SDS) [21], Zung self-rating anxiety scale (Zung/SAS) [22], state-trait anxiety inventory for children (STAIC) [23], and penile perception score (PPS)[24]), and the International Index of Erectile Function-5 (IIEF-5) [25]. The duration of follow-up extended from the immediate postoperative window to more than 10 years after surgery. The detailed characteristics of the included studies are presented in Table 1.

**Table 1:** Characteristics of individual studies included in the systematic review of psychological and behavioral outcomes after hypospadias repair. ADHD: Attention-Deficit/Hyperactivity Disorder; ASD: Autism Spectrum Disorder; CBCL: Child Behavior Checklist; CDI: Children's Depression Inventory; CFS: Children's Fear Scale; CRIES-13: Children's Revised Impact of Event Scale-13; FU: Follow-up; GEDS: Genital Examination Distress Scale; HOPE: Hypospadias Objective Penile Evaluation; HOSE: Hypospadias Objective Scoring Evaluation; HRQoL: Health-Related Quality of Life; IIEF-5: International Index of Erectile Function-5; OCD: Obsessive-Compulsive Disorder; PedsQL: Pediatric Quality of Life Inventory; PGWBI: Psychological General Well-Being Index; PHBQ: Post Hospitalization Behavior Questionnaire; PPPS: Pediatric Penile Perception Score; PPS: Penile Perception Score; PSC-17: Pediatric Symptom Checklist-17; PTSD: Post-Traumatic Stress Disorder; QoL: Quality of Life; RCADS: Revised Child Anxiety and Depression Scale; SDQ: Strengths and Difficulties Questionnaire; SPPA: Self-Perception Profile for Adolescents; STAIC: State-Trait Anxiety Inventory for Children; YSR: Youth Self-Report; Zung SAS: Zung Self-Rating Anxiety Scale; Zung SDS: Zung Self-Rating Depression Scale, ID: Intellectual disability.

Author (Country)	Study design	Age at operation	Total patients	Outcome assessed	Key findings	Assessment tools	Hypospadias Severity
Mureau et al., 1997 [9] (Netherlands)	Cross-sectional comparative	Childhood; FU 9–18 & 18–38 yrs	189	Social anxiety, self-confidence, emotional/behavioral	Body image concerns; generally no worse psychosocial outcomes vs. controls	CBCL and YSR [16], structured interviews	Mixed (all types)
Duarsa et al., 2019 [36] (Indonesia)	Case-control	1–12 yrs	203	Psychosocial disorders, PTSD	29/203 had psychosocial impairment; PTSD not significant	PSC-17 [26], HOPE [27], GEDS [28], CRIES-13 [29]	Mixed (all types)
Jones et al., 2009 [37] (Australia)	Prospective cohort	<5 vs >5 yrs	55	Psychosexual adjustment	Genital dissatisfaction linked to poor psychosexual outcomes	HOSE [30], QoL, self-report	Mixed (all types)
Gulseth et al., 2025 [38] (Norway)	Cross-sectional with controls	Childhood; FU age 16	117 + 61	Mental health, low self-esteem	16% borderline/case; proximal cases less satisfied	SDQ [18], PedsQL [17], SPPA [31], PPPS [24]	Mixed; proximal subgroup
Türk et al., 2013 [39] (Turkey)	Prospective intervention	Childhood	30	Hospital anxiety	Video follow-up reduced anxiety significantly	CFS [32]	Not specified
Örtqvist et al., 2019 [40] (Sweden)	Registry-based cohort	Childhood (1959–1994)	167	Psychiatric morbidity	No higher risk vs. controls	Registry + psych scales	Mixed (registry)
Schönbucher et al., 2008 [10] (Switzerland)	Cross-sectional	Childhood; FU 7–17 yrs	68 + 68	Psychosexual development	Comparable to controls; penile self-perception issues during puberty	PPPS [24], Gender-Role	Mixed (all types)
Andersson et al., 2018 [11] (Sweden)	Prospective (proximal)	FU 14–25 yrs	33	Psychosexual, body image	39% dissatisfied with penile length; intimacy concerns	PGWBI [33], Body-Esteem, PPS [24]	Proximal only
Butwicka et al., 2015 [41] (Sweden)	Nationwide registry	Childhood	9,262	Neurodevelopmental (ASD, ADHD, ID)	Increased risk of ASD, ADHD, ID	National registries	Mixed (registry)
Luo et al., 2019 [42] (China)	Prospective cohort	2–12 yrs	177	Postoperative behavioral changes	60% negative changes at 2 wks, 46% at 1 mo	PHBQ [34]	Mixed (all types)
Miller & Grant, 1997 [43] (USA)	Retrospective cohort	Childhood	56	Psychosexual development	Mixed outcomes; some anxiety in adulthood	Clinical interview	Proximal / severe
Wang et al., 2009 [44] (China)	Retrospective cohort	<10, 10–18, >18 yrs	130 + 50	Anxiety, depression, psychosexual	Higher anxiety/depression in late repairs	Zung SDS/SAS [21,22], IIEF-5 [25]	Mixed (all types)
Eray et al., 2005 [45] (Turkey)	Retrospective comparative	<30 mo vs >30 mo	40	Depression, anxiety	No significant difference	CDI [19], STAIC [23]	Mixed (all types)
Weber et al., 2009 [46] (Switzerland)	Cross-sectional	<18 mo vs >18 mo	77	Adjustment, QoL, gender-role	No significant difference	CBCL [16], PPPS [24], HRQoL	Mixed (all types)
Aydın et al., 2024 [47] (Turkey)	Retrospective comparative	<2 yrs vs >2 yrs	70 + 70	Anxiety, depression, OCD, somatic	OCD higher in >2 yrs; otherwise no worse	SDQ [18], RCADS [20], ARI [35], PPS [24]	Distal only

*Risk of Bias* 

Methodological appraisal with the Newcastle-Ottawa instrument produced scores that ranged across the included studies from low to good. Across the cohort investigations, selection of participants and ascertainment of outcomes were generally satisfactory; however, comparability of comparison groups represented a recurring methodological weakness. A study-by-study summary of the risk-of-bias evaluation is provided in Table 2.

**Table 2:** Risk of bias assessment summary using the Newcastle-Ottawa Scale. Newcastle-Ottawa Scale (NOS) for non-randomized studies. Scores are presented as integers for each domain: Selection (maximum 4), Comparability (maximum 2), and Outcome (maximum 3), giving a theoretical maximum of 9. Quality grading: total score ≥7 = High methodological quality; 4–6 = Moderate quality; <4 = Low quality.

Author (Year)	Study design	Selection (0–4)	Comparability (0–2)	Outcome (0–3)
Mureau et al., 1997 [9]	Cross-sectional comparative	2	1	2
Duarsa et al., 2019 [36]	Case-control	2	2	2
Jones et al., 2009 [37]	Prospective cohort	4	2	3
Gulseth et al., 2025 [38]	Cross-sectional with controls	3	2	2
Türk et al., 2013 [39]	Prospective cohort	2	1	2
Örtqvist et al., 2019 [40]	Registry-based cohort	4	2	2
Schönbucher et al., 2008 [10]	Cross-sectional	3	1	2
Andersson et al., 2018 [11]	Prospective cohort	4	2	2
Butwicka et al., 2015 [41]	Nationwide registry cohort	4	2	2
Luo et al., 2019 [42]	Prospective cohort	3	1	2
Miller & Grant, 1997 [43]	Retrospective cohort	3	1	2
Wang et al., 2009 [44]	Retrospective cohort	3	2	2
Eray et al., 2005 [45]	Retrospective cohort	2	1	2
Weber et al., 2009 [46]	Cross-sectional	2	1	2
Aydın et al., 2024 [47]	Retrospective comparative	3	2	2

*Psychological and Behavioral Outcomes* 

A total of 15 reports examined psychological and psychosexual sequelae after hypospadias correction, employing a range of methodological designs and evaluation strategies. In an earlier study, Miller and Grant tracked adult men born with severe perineoscrotal hypospadias and documented enduring urinary as well as ejaculatory impairment, though psychiatric morbidity was relatively modest in their cohort. Prospective evaluations by Eray and colleagues from Turkey and Weber and coworkers in Switzerland identified that, although postoperative emotional symptoms, including anxiety and depressive features, were present, the magnitude of these outcomes did not appear to differ meaningfully according to the age at which the operation had been performed, offering little support for a psychological benefit from earlier intervention. In a more recent Turkish series, Aydın and colleagues compared boys treated for distal hypospadias with healthy controls and noted a generally lower burden of depressive and anxious symptoms, while obsessive-compulsive features were more prevalent among children whose operation took place beyond two years of age [13,21-23,30].

Large-scale population studies from Sweden by Butwicka et al. and from China by Luo and coworkers underscored increased susceptibility to both neurodevelopmental and psychiatric conditions among operated patients, with notable elevations in the risk of ADHD and ASD diagnoses. Several teams, including Jones et al. in Australia and Andersson et al. in Sweden, documented difficulties with body image, lower self-perceived sexual confidence, and longstanding dissatisfaction with the appearance of the genitalia in a portion of their adolescent and adult subjects. Other single-institution analyses, among them those by Mureau and colleagues in the Netherlands and Wang and colleagues in China, indicated that adequate psychosocial functioning is achievable for many operated patients, although a subset continues to show vulnerability to anxious or depressive symptoms and to reduced self-esteem. Synthesizing this body of literature, although the majority of patients achieve favorable psychosocial trajectories, specific subpopulations, notably those operated for proximal anomalies, those who have undergone repeated surgeries, or those exhibiting negative self-image, may derive particular benefit from intensified psychological monitoring and continued follow-up extending through adolescence into adult life [9,16-23].

*Synthesis of Findings* 

Considering the 15 studies that addressed psychosocial and psychosexual domains as a whole, the majority of operated patients ultimately achieved acceptable long-term adjustment after their corrective surgery. Even so, a persistent subgroup continued to demonstrate susceptibility to unfavorable endpoints, encompassing dissatisfaction with body appearance, diminished sexual self-assurance, and clinical features of anxiety, depression, or neurodevelopmental disorder. A consistent psychological benefit from earlier operation could not be established across reports, although a portion of the available evidence indicated reduced obsessive-compulsive symptoms and better body satisfaction among children whose repair occurred before two years of age. Registry-derived cohorts tended to emphasize a heightened burden of psychiatric morbidity, while clinical series of smaller scale more often surfaced issues related to genital self-image and emotional adjustment, particularly in patients with proximal lesions or those who had undergone several operations.

*Certainty of Evidence* 

Applying the GRADE framework, the confidence that could be placed in the body of evidence varied between low and moderate across outcomes. The principal reasons for downgrading were methodological limitations inherent to observational designs and reliance on self-reported measures, inconsistencies between cohorts, and indirectness arising from differences in age categorization and outcome measurement approaches across studies. Detailed certainty ratings for each outcome are shown in Table 3.

**Table 3:** GRADE evidence profile for psychological effects and behavioral outcomes after hypospadias repair. GRADE certainty of evidence is denoted by filled and open circles. ⊕⊕⊕⊕ HIGH (very confident that the true effect lies close to the estimate); ⊕⊕⊕○ MODERATE (moderately confident; true effect likely close to the estimate but possibly substantially different); ⊕⊕○○ LOW (limited confidence; true effect may be substantially different from the estimate); ⊕○○○ VERY LOW (very little confidence; true effect is likely to be substantially different). ADHD, attention-deficit/hyperactivity disorder; ASD, autism spectrum disorder; OCD, obsessive-compulsive disorder.

Outcome	Studies (patients)	Findings	GRADE certainty	Reasons for downgrade
Psychosexual development	6 studies (~800)	Generally comparable to controls; dissatisfaction with penile appearance linked to worse outcomes	Low ⊕⊕○○	Risk of bias (observational, self-report); inconsistency; indirectness
Behavioral changes	4 studies (~350)	Postoperative problems in 30–60%; younger age and multiple procedures linked to worse outcomes	Low ⊕⊕○○	Risk of bias; inconsistency; imprecision
Anxiety and depression	4 studies (>12,000)	Registry: increased risk of ADHD, ASD, depression/anxiety. Cohort studies: no significant difference; OCD higher if surgery >2 yrs	Low to Moderate	Inconsistency (registry vs cohort); upgraded for large sample size

Discussion

Reconstruction of hypospadias continues to rank among the more demanding pediatric urological procedures [3]. The objective of an ideal repair is to achieve consistently low complication rates while producing a penis that functions normally for both micturition and erection, and that has an acceptable cosmetic appearance [1,3]. Debate over the most suitable age for operation has persisted for many years, accompanied by evolving recommendations. Earlier work suggested that surgical success was not influenced by age [7,8], whereas more contemporary comparative studies have indicated that younger patients may experience fewer postoperative complications [48,49]. Putative advantages of early operation include easier postoperative care, often facilitated by the use of double-nappy regimens, a reduced incidence of acute urinary retention, possibly improved tissue healing, and a lower psychological imprint; however, the relationship between operative age and surgical complication rates remains contested [7,8,48,49].

In contrast to most prior systematic appraisals, the present synthesis was specifically directed at the psychological repercussions of when surgery is performed. Our results suggest that, for the majority of operated boys, long-term psychosocial functioning is acceptable, yet a recurring fraction continues to report ongoing problems, including anxious symptomatology, depressive features, obsessive-compulsive tendencies, and concerns about body appearance. While a subset of reports has linked operation at younger ages to better psychosexual endpoints, such as enhanced satisfaction with body image and a reduced obsessive-compulsive symptom load [7,47], other investigations have failed to find meaningful differences attributable to age at operation [45,46]. The divergent conclusions are most plausibly explained by heterogeneity across studies in methodology, choice of measurement tools, and the cultural settings in which they were conducted. Furthermore, large-scale registry analyses have documented an increased burden of psychiatric and neurodevelopmental conditions in this patient group independent of operative age, raising the possibility that constitutional biological or genetic predispositions may also contribute [40,41].

Within lower-resource healthcare settings, children with hypospadias are often brought to medical attention at older ages than their counterparts in better-resourced countries. Restricted access to specialty care, gaps in caregiver awareness, and economic limitations contribute to this delay, and corrective procedures are commonly carried out at whatever age the child eventually reaches a treating center, often beyond four years of age. Indicators of socioeconomic context, for example, geographic location of the household and parental schooling, correlate with both the timing of operation and the probability that repair will be postponed [6]. These observations probably reflect underlying differences in how healthcare systems are organized and how readily specialist pediatric urology services can be reached. Meatal location also influences the diagnostic trajectory: anomalies positioned more proximally are typically detected sooner owing to coexisting voiding problems, while severe forms may trigger earlier referral because of perceived concerns regarding sexual differentiation or the ambiguous appearance of the genitalia. Repair carried out early is commonly considered advantageous because the penis is smaller with more pliable tissue at that age, the child has limited cognitive awareness of the procedure, and both cosmetic and technical demands of the operation may be reduced [51]. In contrast, postponed surgery has, in certain series, been associated with thicker scar tissue, greater intraoperative difficulty, and higher rates of postoperative complications [4,7,8,50].

Notably, studies restricted to proximal hypospadias, such as Andersson et al. [11] and the proximal subgroup in Gulseth et al. [38], consistently reported worse psychological and body image outcomes compared to mixed or distal cohorts, underscoring the independent contribution of anatomical severity to long-term psychosocial adjustment and reinforcing the need for severity-stratified analyses in future prospective investigations. Several biological and neurodevelopmental mechanisms may underlie the relationship between operative age and psychological outcomes. Young children undergoing surgery during the first 6-18 months of life are at a stage of rapid neurological maturation, during which the brain retains considerable plasticity, and explicit episodic memory formation is developmentally immature. As a result, procedural trauma incurred at this age is less likely to be consciously encoded, potentially limiting the psychological imprint of surgical experience. In contrast, children operated at older ages possess an emerging sense of body awareness and genital self-perception, rendering them more susceptible to psychological distress, negative body image, and social comparison effects. From an endocrine perspective, androgen exposure, heightened during the mini-puberty of infancy and again at puberty, influences penile tissue architecture and the psychological processing of genital difference. Furthermore, early repair allows the child to enter the preschool years with restored anatomy, which may support the formation of an undisrupted body schema and healthier psychosexual identity development. These mechanistic considerations, supported by evidence from Bermudez et al. [51], Aydın et al. [47], and Jones et al. [37], provide a plausible biological framework for the clinical observation that earlier surgical intervention may confer psychological advantages, even when aggregate study outcomes remain inconclusive.

Key strengths of the current work include the breadth of the literature search, screening performed independently by two reviewers, and a structured evaluation of risk of bias via the Newcastle-Ottawa instrument, with the strength of the evidence further graded according to GRADE. A number of limitations nonetheless warrant explicit consideration. To begin with, most included reports were observational in nature, and only a small subset provided granular stratification according to severity of the anomaly, type of surgical procedure used, or operating surgeon’s experience, all factors known to influence results. Second, the psychological endpoints assessed across studies showed marked variability with respect to study design and the instruments selected, which prevented meta-analytic pooling and constrained the synthesis to a descriptive interpretation. Third, statistical evaluation of publication bias could not be performed because the number of studies addressing any single outcome fell below the conventional threshold of 10. Fourth, formal inter-rater agreement statistics (e.g., Cohen’s kappa) were not calculated, which represents a limitation in the transparency of the review process; future reviews should incorporate kappa calculation at both the title/abstract and full-text screening stages. Fifth, restriction of the search to English-language publications constitutes a potential source of language bias, as relevant studies conducted and published in non-English-speaking countries, particularly from East Asian, Middle Eastern, and Latin American settings where hypospadias surgical volumes are considerable, may have been inadvertently excluded; future systematic reviews should consider incorporating multilingual search strategies to mitigate this limitation.

Subsequent multicenter prospective work employing standardized outcome reporting frameworks, careful stratification by anatomical severity, and incorporation of validated psychometric measures will be required to better define how operative timing affects both physical and psychosocial endpoints. Extended follow-up reaching into adolescence and adult life is of particular value, given that effects on body image, sexual functioning, and psychiatric outcomes may not become apparent until later developmental stages.

## Conclusions

Overall, psychological trajectories following hypospadias correction are generally favorable, yet a recurring subgroup develops anxiety, depression, or body image dissatisfaction, and the effect of operative timing on these outcomes remains inconclusive across available studies. Psychosocial monitoring must therefore be considered an integral component of postoperative care rather than an optional adjunct. A fundamental barrier to progress is the absence of standardized outcome measures: at least 20 distinct instruments were identified across the 15 included studies, encompassing the CBCL, SDQ, RCADS, PPPS, PedsQL, Zung SDS/SAS, CDI, STAIC, PPS, YSR, PGWBI, and HOSE, precluding cross-study comparability and meta-analytic pooling. Future multicenter prospective research should prioritize the adoption of a consensus-derived core outcome set incorporating validated measures of penile self-perception, psychiatric screening, and disease-specific quality of life, assessed at standardized developmental milestones from early childhood through adulthood.

## Appendices

Supplementary Appendix A

Complete Database-Specific Search Strategies

The following search strings were executed in May 2025 across four electronic databases. All searches were conducted without date restriction and were limited to English-language publications. Boolean operators, field tags, and controlled vocabulary terms are presented exactly as applied in each database.

A.1 PubMed (National Library of Medicine)

Date of search: May 2025. Results: 380 records.

((hypospadias[MeSH Terms] OR hypospadias[tiab] OR hypospadias repair[tiab] OR hypospadias surgery[tiab] OR urethroplasty[tiab]) AND (patient age[tiab] OR age at surgery[tiab] OR age at repair[tiab] OR timing of surgery[tiab] OR timing of repair[tiab] OR timing of hypospadias repair[tiab] OR operative age[tiab] OR age at operation[tiab]) AND (psychological[tiab] OR psychosocial[tiab] OR psychosexual[tiab] OR psychiatric[tiab] OR behavioral[tiab] OR behavioural[tiab] OR cognitive development[tiab] OR genital awareness[tiab] OR emotional development[tiab] OR psychosexual development[tiab] OR mental health[tiab] OR anxiety[tiab] OR depression[tiab] OR body image[tiab] OR self-esteem[tiab] OR quality of life[tiab] OR neurodevelopmental[tiab] OR ADHD[tiab] OR autism[tiab] OR obsessive-compulsive[tiab])) AND (English[lang])

A.2 Embase (Elsevier)

Date of search: May 2025. Results: 312 records.

('hypospadias'/exp OR 'hypospadias':ti,ab OR 'hypospadias repair':ti,ab OR 'hypospadias surgery':ti,ab OR 'urethroplasty':ti,ab) AND ('patient age':ti,ab OR 'age at surgery':ti,ab OR 'age at repair':ti,ab OR 'timing of surgery':ti,ab OR 'timing of repair':ti,ab OR 'operative age':ti,ab OR 'age at operation':ti,ab) AND ('psychology'/exp OR 'psychological':ti,ab OR 'psychosocial':ti,ab OR 'psychosexual':ti,ab OR 'psychiatric':ti,ab OR 'behavioral':ti,ab OR 'behavioural':ti,ab OR 'mental health':ti,ab OR 'anxiety'/exp OR 'anxiety':ti,ab OR 'depression'/exp OR 'depression':ti,ab OR 'body image':ti,ab OR 'self esteem':ti,ab OR 'quality of life':ti,ab OR 'neurodevelopmental':ti,ab OR 'attention deficit hyperactivity disorder'/exp OR 'autism spectrum disorder'/exp OR 'obsessive compulsive disorder'/exp) AND [english]/lim

A.3 ScienceDirect (Elsevier)

Date of search: May 2025. Results: 198 records.

(hypospadias OR "hypospadias repair" OR "hypospadias surgery" OR urethroplasty) AND ("patient age" OR "age at surgery" OR "age at repair" OR "timing of surgery" OR "timing of hypospadias repair" OR "operative age") AND (psychological OR psychosocial OR psychosexual OR psychiatric OR behavioral OR behavioural OR "mental health" OR anxiety OR depression OR "body image" OR "self-esteem" OR "quality of life" OR neurodevelopmental OR "cognitive development" OR "genital awareness" OR "emotional development" OR "psychosexual development")

A.4 Cochrane Library (Wiley)

Date of search: May 2025. Results: 55 records.

(hypospadias OR "hypospadias repair" OR urethroplasty) AND ("patient age" OR "age at surgery" OR "timing of surgery" OR "operative age" OR "age at repair") AND (psychological OR psychosocial OR psychosexual OR psychiatric OR behavioral OR "mental health" OR anxiety OR depression OR "body image" OR "quality of life" OR neurodevelopmental)

Total records retrieved across all databases (before deduplication): 945 (PubMed: 380; Embase: 312; ScienceDirect: 198; Cochrane Library: 55). Records screened at title/abstract: 945. Records excluded at title/abstract screening: 913. Full-text articles assessed for eligibility: 32. Full-text articles excluded: 17 (irrelevant study design, n=9; not expected predictor/outcome, n=8). Studies included in final synthesis: 15.
