# Correction: Psychological Outcomes After Hypospadias Repair: Does Age at Surgery Matter? A Systematic Review

**DOI:** 10.7759/cureus.c453

**Published:** 2026-06-22

**Authors:** Randy Fauzan, Irfan Wahyudi, Gerhard Reinaldi Situmorang, Arry Rodjani, Putu Angga Risky Raharja

**Affiliations:** 1 Urology, Rumah Sakit Umum Pusat Nasional Dr. Cipto Mangunkusumo, Jakarta, IDN

This article has been corrected to address several reference and citation discrepancies. The following errors were observed:

- Several tools/scores/checklists were not referenced. 
- The existing citations were not ordered sequentially.
- Some studies were cited, but not included in the reference list.

As a result, multiple references and citations have been added. Additionally, several stylistic and language-related errors have also been corrected.

